# Democratizing ownership and participation in the 4th Industrial Revolution: challenges and opportunities in cellular agriculture

**DOI:** 10.1007/s10460-021-10237-7

**Published:** 2021-08-24

**Authors:** Robert M. Chiles, Garrett Broad, Mark Gagnon, Nicole Negowetti, Leland Glenna, Megan A. M. Griffin, Lina Tami-Barrera, Siena Baker, Kelly Beck

**Affiliations:** 1 Department of Agricultural Economics, Sociology, and Education, Department of Food Science, Rock Ethics Institute, Penn State University, University Park, USA; 2 Department of Agricultural Economics, Sociology, and Education, Penn State University, Armsby Bldg, University Park, PA 16801 USA; 3 Department of Communication and Media Studies, Fordham University, Faculty Memorial Hall, 2546 Belmont Ave, Bronx, NY 10458 USA; 4 Animal Law & Policy Program, Harvard Law School, 1607 Massachusetts Avenue, Cambridge, MA 02138 USA; 5 Department of Agricultural Economics, Sociology, and Education, International Agriculture and Development Graduate Program, Penn State University, Armsby Bldg, University Park, PA 16801 USA; 6 Department of Agricultural Economics, Sociology, and Education, Department of Economics, Penn State University, Armsby Bldg, University Park, PA 16801 USA

**Keywords:** Food and agricultural ethics, Science, technology, and society, Cellular agriculture, Social inequality, Political economy of agriculture, Digital agriculture, Rural sociology

## Abstract

The emergence of the “4th Industrial Revolution,” i.e. the convergence of artificial intelligence, the Internet of Things, advanced materials, and bioengineering technologies, could accelerate socioeconomic insecurities and anxieties or provide beneficial alternatives to the status quo. In the post-Covid-19 era, the entities that are best positioned to capitalize on these innovations are large firms, which use digital platforms and big data to orchestrate vast ecosystems of users and extract market share across industry sectors. Nonetheless, these technologies also have the potential to democratize ownership, broaden political-economic participation, and reduce environmental harms. We articulate the potential sociotechnical pathways in this high-stakes crossroads by analyzing cellular agriculture, an exemplary 4th Industrial Revolution technology that synergizes computer science, biopharma, tissue engineering, and food science to grow cultured meat, dairy, and egg products from cultured cells and/or genetically modified yeast. Our exploration of this space involved multi-sited ethnographic research in both (a) the cellular agriculture community and (b) alternative economic organizations devoted to open source licensing, member-owned cooperatives, social financing, and platform business models. Upon discussing how these latter approaches could potentially facilitate alternative sociotechnical pathways in cellular agriculture, we reflect upon the broader implications of this work with respect to the 4th Industrial Revolution and the enduring need for public policy reform.

## Introduction

Emerging “4th Industrial Revolution” (4IR) technologies that fuse the “physical, digital, and biological worlds” (Schwab 2016, p. 1) could help to revitalize the contemporary agri-food system, improve social equity, and reduce environmental degradation. They could also do the opposite. Cellular agriculture, a 4IR technology that uses animal cells—or, in some cases, genetically modified yeast—to grow meat, dairy, egg, seafood, and plant components in vitro is an exemplar of this existential tension. Although it is not yet widely available as a consumer product, its proponents portray a best-case scenario in which cellular agriculture could reduce land, water, and chemical inputs, minimize greenhouse gas emissions, improve food safety, optimize nutritional attributes, and obviate the need to raise and slaughter animals for food (Cameron and O’Neill 2019; Datar et al. 2016; Specht et al. 2018). However, cellular agriculture could also concentrate ownership and power in the global food system, namely by displacing ranchers, farmers, fishers, and ancillary industries, while also falling short of its environmental promises (Broad 2019; Cosgrove 2017; Hines et al. 2018; Lynch and Pierrehumbert 2019; Tuomisto 2019).

Over the past decade, scholars have explored a broad spectrum of socioeconomic and ethical questions pertaining to this technology (see Chiles 2013; Guthman and Biltekoff 2020; Hocquette et al. 2015; Jönsson 2016; Laestadius 2015; Slade 2018; Broad 2019). However, this scholarship has done little to engage with concepts related to inclusive innovation (Juma 2016; Stilgoe et al. 2013), and therefore has yet to identify mechanisms that might facilitate more (or less) just and equitable development in this sector. Scholarship in the political economy of agriculture provides a logical starting point for such an inquiry, as it has demonstrated how supply chain configurations, public and private governance systems, and the social construction of technology affect consumption patterns and livelihoods. Perhaps above all, this literature showcases the importance of understanding food-system ownership and stakeholder participation at a structural level (Howard 2016). However, as noted by Gibson-Graham (2006), there is a tendency for many political economy scholars to portray the capitalist economy as monolithic and incessant. Large transnational firms have gained powerful oligopolistic market control with global influence that will likely persist. However, their continued dominance is not a foregone conclusion, as there are alternative social organizations of economic activities that could become more prominent alongside 4IR innovations. This is important because, as previous industrial revolutions have revealed, the potential socioeconomic and ecological benefits of technological innovations are contingent on the social organization of their production and distribution. Industrial revolutions thus present the possibility for social reorganization that could democratize ownership, broaden political-economic participation, and reduce environmental harms (McCarthy and Zald 1977; Rifkin 2014; Schwab 2016).

We explore these issues by investigating how alternative economic models might facilitate a process of inclusive innovation in cellular agriculture, and by extension, provide a template for other 4IR technologies. In what follows, we first engage with and extend the political economy of agriculture literature by examining how digitalization is reshaping traditional forms of ownership and participation in the agri-food system. We then discuss our empirical investigation of this phenomenon, which has been guided by two basic research questions: (I) How do players in the cellular agriculture community understand its current economic trajectory? (II) What types of alternative sociotechnical pathways could be used to develop cellular agriculture? Our approach to these questions involved multi-sited ethnographic research of the cellular agriculture community and alternative economic organization events. After reviewing our findings from these two respective areas of inquiry, we synthesize our results by discussing a unique sociotechnical pathway that could facilitate inclusive innovation in this field. We conclude the paper by reflecting upon the broader implications of this work, specifically with respect to the 4IR, alternative food movements, food tech justice, and public policy.

## The 4th Industrial Revolution and cellular agriculture in political-economic context

Historically, while the appropriation and substitution of raw natural materials for industrial products has resulted in innovative solutions for agricultural challenges (Lusk 2016), it has also concentrated political and economic power in the hands of the largest farmers, input providers, processors, and retailers (Goodman et al. 1987; Howard 2016). Horizontal/vertical integration, intellectual property rights, financialization, and private governance have further squeezed market share from small producers (Carlson 2018; Lawrence 2017; Marsden et al. 2019; Patel and Moore 2017; Pimbert et al. 2001). Proprietary-driven innovation strategies have become so prominent that even public universities have sought to attract more private investments and intellectual property protections (Brandl and Glenna 2017).

Agricultural scientists and large agribusiness actors have also changed the depth and composition of social participation in the agricultural system. There are fewer farms, experiential knowledge of farming has been largely displaced by technocratic expertise, research is driven by an emphasis on proprietary and productivist technologies, communities are increasingly disconnected from traditional foodways, consumers and workers are “deskilled” (Jaffe and Gertler 2006), and civic engagement is sporadic. In the seed and agricultural sectors, intellectual property licensing has led to greater market concentration and less competition (Fuglie and Toole 2014), and studies suggest that intellectual property protections and lawsuits may inhibit certain types of university research (Baker et al. 2017; Lei et al. 2009; Glenna et al. 2015).

While the debate over industrial agriculture and intellectual property continues, a new era of technological disruption has already begun. The convergence of digital technology with materials science, biology, and other fields is now “evolving at an exponential rather than linear pace” (Schwab 2016, p. 1). In agriculture, artificial intelligence is significantly reducing the use of labor (Carolan 2018; Rotz et al. 2019a); embedded sensors, drones, and cloud/edge computing are advancing the field of “smart” agriculture (Rotz et al. 2019b); traditional manufacturing companies like John Deere are reinventing themselves as “technology companies” (Carolan 2018); Walmart is deploying blockchain to enhance traceability in supply chains (Corkery and Popper 2018); 3D printers are being used to decentralize and personalize food manufacturing (Nachal et al. 2019); and synthetic biology is dramatically accelerating plant and animal breeding (Pixley et al. 2019). These shifts are being further accelerated in the aftermath of the Covid-19 pandemic (Chiles 2020). Outside of academic research, 4IR innovations have primarily emerged from the conventional playbook of commercial technology development, i.e. leveraging private investments, intellectual property, and/or government subsidies (Baker et al. 2017). If concerned stakeholders do not proactively engage with these developments, the deployment of digital technologies can potentially “lock-in” institutional arrangements and build path dependencies (Carolan 2020a).

Trailblazing work on digitalization and agri-food systems has been done by Wolf and Wood (1997), Kloppenburg (2010, 2014), Carolan (2015, 2017a, b, 2018), Schneider et al. (2017), and others, but the political economy of agriculture as a whole has been slow to recognize the profoundly disruptive potential of the 4IR. The transformative impact of the 4IR on global agri-food systems is now virtually impossible to ignore, and interest in this area has grown significantly in just the past two years (see Rotz et al. 2019a, b; Bowen and Morris 2019; Burton 2019; McMichael 2020; Broad 2020b; Barrett and Rose 2020; Carolan 2020b; Clapp and Ruder 2020; Comi 2020; Fraser 2020; Hansen et al. 2020; Marshall et al. 2020; Oncini et al. 2020; Rose et al. 2021). In the digital platform era, optimizing efficiencies through horizontal/vertical integration and supply-side economies of scale (e.g. Tyson Foods), dictating strict contractual terms to suppliers (e.g. Walmart), or providing a uniform customer experience (e.g. McDonalds) is no longer the only logical strategy for accumulation. Now, “with platforms, the assets that are hard to copy are the community and the resources its members own and contribute, be they rooms or cars or ideas and information. In other words, the network of producers and consumers is the chief asset” (Van Alstyne et al. 2016). Here, minimal-input, highly efficient, decentralized, and personalized 4IR technologies that produce at near “zero marginal cost” (Rifkin 2014) could eventually replace twentieth century corporate strategies.

Cellular agriculture embodies the 4IR by integrating digital, material, and biological technologies, specifically computer science, biopharma, tissue engineering, and food science (Datar et al. 2016). Cell-based meat production involves collecting immortalized cell lines, maturing them in a nutrient-rich growth media, seeding them on biomechanical scaffolding in a proliferation bioreactor, and then harvesting the edible muscle tissue. Production processes for cell-based meat have been under development since the turn of the millennium, initially in the form of goldfish “fillets” (Benjaminson et al. 2002), bioengineering exhibits (Catts and Zurr 2008), and, more recently, through venture-capital-funded startups and applied research in product development (Stephens et al. 2019). Policymakers in the U.S. and globally are still deciding how to regulate these products, as many of the underlying technologies are still under development and/or undisclosed for proprietary reasons (see the Good Food Institute’s annual *State of the Industry Report* for updates). At the time of this writing, consumers in select geographies are just beginning to have access to some cellular agriculture products, but in the US and throughout most of the world, the industry remains in a pre-market development phase (Noyes 2020).

Given the asymmetrical distribution of benefits and harms from previous industrial technologies, the political economy of agriculture scholarship has generally been skeptical of technological solutions for contemporary food system challenges (Scott 2011). There is widespread concern, therefore, that cellular agriculture could accelerate the concentration of wealth and diminish overall participation in agriculture through “de-agrarianization” (Hebinck 2018), all while offering fewer environmental and public health benefits than promised (Bowen and Morris 2019; Broad 2019; Lynch and Pierrehumbert 2019; Perls 2018). These concerns merit further exploration and deliberation. There could be a significant centralization of power, for example, if a single company perfects a market-ready product first, or if that company is absorbed by a larger corporation. Due to the longevity of patent protections, there might be little incentive for new product development (Clancy and Moschini 2017). Competing industry principles of disruption, transparency, and secrecy may prevent interested publics from fully assessing the technology’s consequences or holding its proponents accountable (Guthman and Biltekoff 2020). A massive disruption to the livestock industry would moreover have profound socioeconomic impacts on a host of different agricultural stakeholders (Ilea 2009). Many ranchers, for example, are self-employed, employ lower skilled workers, and manage small herds—79% of beef cattle farmers own fewer than 49 animals (USDA NASS 2019). Taken together, there is good reason to be skeptical of techno-utopian rhetoric (Broad 2020a; Jönsson 2016).

At the same time, however, it is important not to adopt a pre-determined perspective that assumes agri-food technologies are inherently linked to inequity and exclusion. Braveman and Gruskin (2003) note that equity—an absence of systemic disparities in health and well-being between groups with different levels of social power—can be realized through improved social processes. Juma (2016) makes a similar observation with respect to inclusive innovation, a process through which diverse public engagement is fostered and local capabilities are built, particularly among historically excluded communities. While this is rarely a priority for profit-driven transnational corporations, it can nonetheless be advanced through existing institutional frameworks such as public oversight, joint ventures and technology partnerships, and equitable management of intellectual property (Juma 2016). Related frameworks have emphasized that incorporating dimensions of anticipation, reflexivity, inclusion, and responsiveness into the early development process can promote responsible innovation that “takes care of the future through collective stewardship of science and innovation in the present” (Stilgoe et al. 2013, p. 1571). The growing field of responsible innovation and research is built upon a belief that scientific and technological advances can be productively embedded in society through a, “transparent, interactive process by which societal actors and innovators become mutually responsive to each other with a view on the (ethical) acceptability, sustainability and social desirability of the innovation process and its marketable products (von Schomberg 2011, p. 9). Similarly, for Djelic and Quack (2007), new and alternative “path generation” can take shape with the aggregation of multiple decision points and critical junctures over time where “legacies play an important but non-deterministic role” (Djelic and Quack 2007, p. 167).

Although digitalization and platformization have disproportionately benefitted the world’s largest companies, they have simultaneously expanded how food system workers, small producers, citizen consumers, food justice activists, and scholars can participate in collective action and institutional decision-making (see Akom et al. 2016; Carolan 2017a, b; Schneider et al. 2017). Increasing democratic participation in regulatory agencies and economic institutions, for example, can enhance problem solving by reducing knowledge gaps and building trust between citizens, employees, and executives (Dewey 1991; Dryzek 2005; Glenna and Mitev 2009; Katz and Light 1996). The ability to harness new forms of digital participation moreover allows savvy organizations to unleash the power and creativity of the crowd, improve transparency, and make institutions more accessible to ordinary people (Heimans and Timms 2018). Similarly, alternative economic organizations, like cooperatives, can create “inherent structural ties to the local” (Mooney 2004, p. 96) that promote innovation, flexibility, community economic development, and a long-term focus (Glenna and Mitev 2009; Merrett and Walzer 2004).

With cellular agriculture at a moment of nascent if increasingly rapid technological and market expansion, this is a critically important moment for engagement on issues of responsible and inclusive innovation (see also Juma 2016; Lele and Goswami 2017; Lewis 2017; Stilgoe et al. 2013). Given the increasing recognition that the dominant sociotechnical regime results in a skewed distribution of benefits and harms, we sought to explore whether cellular agriculture, and the 4IR more broadly, is being (or could be) developed and diffused through inclusive ownership and participation strategies that offer the potential for equitable outcomes.

## Data and methods

For this project, we conducted multi-sited and online ethnographic research (see Burawoy 2000; Gille and Riain 2002; Kozinets 2015; Marcus 1995) of cellular agriculture and alternative economic organization events. We studied conference events because they effectively serve as proxies for circulating novel discourses, strategizing, innovating, collaborating, and sharing concerns in a hyperconnected, globalized society (see Garsten and Sörbom 2018). In cellular agriculture, conference events “have functioned to both bring people together and symbolically mark new stages in the field’s development” (Stephens et al. 2019, p. 4). When we were unable to do the fieldwork in person, we conducted what Kozinets (2015) refers to as online ethnographies of conference websites, agendas, speakers, sponsors, videos, slides, transcripts, and blog posts. This provided us with additional data on the communities of practice and stakeholder networks in cellular agriculture and alternative economic organizations.

Although several of the events were hosted by organizations that deliberately sought to democratize ownership and participation in the 4IR, others were more agnostic on this goal. Our primary objective was to sample across a broad range of perspectives and sociotechnical pathways through which cellular agriculture could be pursued. In constructing a purposive sample of possible events to target, we first drew on relevant peer-reviewed literatures, business journals, and the collective experiences of an interdisciplinary research team that included scholars with backgrounds in rural sociology, economics, communication, agricultural science, and legal studies. Team brainstorming sessions were then used to inductively select sites of study (see Table 1). We also used these meetings to reflect upon and share how our own respective standpoints, assumptions, blind spots, and values shaped our approach to the fieldwork.

**Table 1:** Data collection at cellular agriculture and alternative economic organization events

Online events	Date	Location
World Agri-Tech Innovation Summit	3/19/19–3/20/19	San Francisco, CA
Good Food Expo	3/22/19–3/23/19	Chicago, IL
National Center for Employee Ownership 2019 Conference	4/9/19–4/11/19	Pittsburgh, PA
Protein Trends and Technologies Seminar	5/21/19–5/22/19	Itasca, IL
SB19—Sustainable Brands	6/3/19–6/6/19	Detroit, MI
Inaugural Meeting of The Cultured Meat Modeling Consortium	6/6/19–6/8/19	Seattle, WA
Plant Based World Conference & Expo	6/7/19–6/8–19	New York, NY
360 of Cooperation: National Conference for Co-ops and Mutuals	6/18/19–6/20/19	Quebec City, QC
Future Food Tech: New York	6/18/19–6/19/19	New York, NY
National Council of Farmer Cooperatives 2019	6/24/19–6/26/19	Washington, DC
Agri Food Innovation Event	6/26/19–6/27/19	Venlo, NL
OPEN 2019—Decentralized Collaboration	6/27/19–6/28/19	London, UK
Open Source Microfactory STEAM camp	6/29/19–7/7/19	Maysville, MO
In Defense of the Commons: Challenges, Innovation and Action (IASC Conference)	7/1/19–7/5/19	Lima, PE
RIPESS Solidarity Economy Europe Gen Assembly	7/5/19–7/6/19	Lyon, FR
2019 MIT Platform Strategy Summit	7/12/2019	Cambridge, MA
Ag Innovation Showcase	9/9/19–9/11/19	Minneapolis, MN
15th Annual Int'l Fair Trade Summit	9/16/19–9/19/19	Lima, PE
Platform Economy Summit Europe	9/16/19–9/19/19	Frankfurt, GER
5th International Scientific Conference on Cultured Meat	10/6/19–10/8/19	Maastricht, NL
2019 Borlaug Dialogue International Symposium	10/16/19–10/20/19	Des Moines, IA

In-Person Events	Date	Location
New Harvest Conference	7/19/19–7/20/19	Cambridge, MA
Open Source Summit 2020	8/21/19–8/23/19	San Diego, CA
Reducetarian Summit	9/27/19–9/29/19	Washington, DC
Good Food Conference	9/4/19–9/6/19	San Francisco, CA
B Corps Champions Retreat	9/16/19–9/18/19	Los Angeles, CA
2019 Co-Op IMPACT Conference	10/2/19–10/4/19	Arlington, VA
SOCAP19 (Social Capital Markets)	10/22/19–10/25/19	San Francisco, CA
CAST (Council for Agricultural Science & Technology) Meeting	10/29/19–10/31/19	Fayetteville, AR
Food Tank Summit NYC	11/1/19–11/2/19	New York, NY
40th Annual Agricultural Law Educational Symposium	11/7/19–11/9/19	Washington, DC
IoT Expo North America 2019	11/13/19–11/14/19	Santa Clara, CA

Although the in-person and online research provided unique types of opportunities for interaction and follow-up, we asked the same research questions and used the same methodological approach for both aspects of the project. For all events, we took note of our initial impressions and assumptions, significant or unexpected activities, patterns, sensory and emotional experiences, key quotations, discourses, local jargon, and participant behaviors, while also pursuing relevant digital threads in greater detail (Emerson et al. 2011; Kozinets 2015; Miles et al. 2014). When we attended the events in person, we further engaged with attendees on the topic of what inclusive innovation for cellular agriculture might look like and how it might be accomplished at the institutional level. Based on prior scholarship in the political economy of agriculture, we asked attendees about the types of organizational models and strategies that they found to be the most inclusive and innovative, whether or not nontraditional organizational models might offer a unique value proposition, the types of customers and potential partners who might be interested, financing options, how scale-up might be achieved, the types of resources that would be needed, which emerging technologies could help to drive innovation in the sector, how intellectual property might be managed, and what types of internal and external challenges would likely be faced. For each event that we studied, both in-person and online, individual researchers inductively sampled on the sessions, participants, and concepts that were most relevant to the core themes of the project (see Becker 1998). We discussed our within-case sampling strategies in team meetings, explained why we chose to focus on certain aspects of the event, and then brainstormed on what we might need to do going forward in order to ensure the saturation of different perspectives.

After we collected the data, we engaged in inductive coding, wrote analytic memos, shared our perspectives in team meetings, and solicited additional feedback from study participants (Emerson et al. 2011; Miles et al. 2014). These interdisciplinary conversations, juxtaposed with relevant literatures, served to triangulate and contextualize our findings (McGrath et al. 1982). Through this process, we identified two critical dimensions for inclusive innovation in cellular agriculture and the 4IR: *ownership*, which refers to the legal entities involved in the development of the industry, and *participation*, which refers to collaboration and stakeholder engagement (both within the industry and with broader publics).

Lastly, our methodology also involved the co-production of alternative sociotechnical pathways for cellular agriculture with our study participants. This approach is widespread in qualitative research, and is manifest across participatory, constructivist, standpoint, and feminist traditions. As noted by Jasanoff (2004, p. 14), the “idiom” of co-production provides a useful framework for analyzing the “relationship between the ordering of nature through knowledge and technology and the ordering of society through power and culture” (cited in Eddens 2019). We accordingly theorized what inclusive ownership and participation in cellular agriculture might look like, and we engaged in reciprocal dialogue with our study participants on different iterations of this concept. The co-production dimension of the research was integrated with the semi-structured interview questions that we asked participants (see above), and it adopted unique shapes and forms based upon the backgrounds of individual participants. This resulted in a hybrid process of scholarship and co-design that emphasized, “reflections, provocations, projections, extrapolations, and anticipations of multiple possible near-future worlds” (Vertesi et al. 2016, p. 181). While we did not want to engage in advocacy, we did want our participants to think creatively about social responsibility, inclusive innovation, and serving the public good. We were transparent about the basic values that animated the project with our interviewees, and we solicited their feedback and suggestions as to their potential meaning(s) and applicability in different institutional and technological contexts. We found that this resulted in rich conversations, built rapport, and stoked interest with the core themes of the project. The co-production aspects of our work are further detailed in the synthesis and reflections section.

## Findings on ownership and participation in cellular agriculture

We found that the field is predominantly (but not exclusively) driven by traditional organizational forms: hierarchical ownership models backed by venture capital and proprietary licensing strategies. These organizational entities empower startups to quickly and effectively participate in the conventional food economy while nonetheless limiting overall collaboration and public engagement in the science itself. From the perspective of our participants, the protection of intellectual property is necessary in order to provide an eventual pay-off to investors and protect their assets from being claimed by rival businesses.

### Ownership

The choice of *legal entity* is foundational to the ultimate social impact of any organization, particularly with respect to how it will be owned and how people might participate within it (Haigh et al. 2015). A legal entity is an association, corporation, partnership, proprietorship, trust, or individual that has legal capacity to enter into agreements or contracts, assume obligations, incur and pay debts, sue and be sued in its own right, and be accountable for illegal activities. The type of legal entity can have significant implications for tax treatment, governance, and operation of an organization, and the amount of control over the organization can also vary depending on how the entity is established. The political economy of agriculture scholarship has long emphasized the importance of legal entities when analyzing social, environmental, and technological change in the global food system (see Chiles et al. 2020; Ransom et al. 1998; Schneiberg et al. 2008; Welsh et al. 2008) and we accordingly used legal entity structures as a gateway towards understanding the landscape of the cellular agriculture industry.

In the cellular agriculture community, a de facto division of labor between legal entities has taken place that effectively mirrors other agricultural and life science sectors: universities conduct basic research; non-profits convene, stimulate, and advocate for the field as a whole; and private companies direct these resources towards commercial applications. The ability of traditional C-corporations and limited liability companies to innovate and grow is undeniable (Haigh et al. 2015), and cellular agriculture entrepreneurs have used these legal entities to accelerate and protect their work. According to the Good Food Institute’s *State of the Industry* report, “27 cell-based meat and seafood companies had publicly announced themselves worldwide by the end of 2018, 11 of which were founded in 2018… [and] 15 have announced that they have raised external funding (Cameron and O’Neill 2019, p. 5; see Table 2).” Eight of these startups, each based in the US, reported a combined $414.5 M in external research and development (R&D) funding.

**Table 2:** Cell-based meat companies

Company	Products (a)	Place and date founded	Total amount raised ($MM)	Most recent funding round
JUST (b)	Cell-culture chicken	San Francisco, CA. 2011	372.5	Series E
Integriculture	Cell-based foie gras	Tokyo, Japan, 2015	2.7	Seed
Mosa Meat	Cultured meat (meatballs, tartare and hamburguer)	Maastricht, Netherlands, 2015	9.0	Series A
SuperMeat	Cultured meat (chicken)	Tel Aviv, Israel, 2015	3.2	Seed
Memphis Meats	Cell-based meatballs and poultry (chicken and duck)	Berkeley, CA. 2015	22.0	Series A
Aleph Farms	Slaughter-free meat (bovine steak)	Rejovot, Israel, 2016	Not disclosed	Seed
Finless Food	Lab-grown fish and seafood	Emery Ville, CA. 2016	3.8	Seed
Wild Type	Lab-grown salmon	San Francisco, CA. 2016	3.5	Seed
Future Meat Technologies	Cell-based meat (chicken)	Jerusalem, Israel, 2017	2.2	Seed
Blue Nalu	Cellular aquaculture and fish	San Diego, CA. 2017	4.5	Seed
Wild Earth	Clean protein dog food	Berkeley, CA. 2017	4.5	Seed
Cubiq Foods	Cell cultured component (chicken fat/Omega3)	Barcelona, Spain, 2018	14.0	Private Equity Buyout
New Age Meat	Clean pork sausage	San Francisco, CA. 2018	0.3	Pre-seed
	Cultured pork muscle-fat product			
Meatable	Lab-grown meat (beef and pork)	Delft, Netherlands, 2018	3.5	Seed
Mission Barns	Duck, chicken, pork (fat component)	Berkeley, CA. 2018	3.5	Seed

(a) Some of the product descriptions were adjusted by the information provided on the companies' web pages. (b) This company is pursuing cell-based meat as one part of a larger business and has not disclosed what portion of its total funding is being devoted to cell-based meat R&D

*Source:* Cameron and O’Neill (2019); reproduced with permission. “For data availability reasons, [the Good Food Institute] report focuses on the 15 companies that have fundraising data available on PitchBook or in another public source (Cameron and O’Neill 2019).”

Several researchers whom we spoke with were either unfamiliar with or skeptical towards the idea of using other types of legal entities to advance the technology. One speaker at New Harvest 2019 (a leading cellular agriculture conference, convened by its namesake non-profit organization), was demonstrably unfamiliar with the concept of a “worker cooperative” when asked. A corporate consultant with whom we spoke at the same event was more explicitly against the idea of alternative legal entities, calling them “too risky,” while supporting the idea of the value-driven and transparent (if nebulously defined) “good corp.” At the same time, however, one engineer with whom we spoke said that a cooperative business for cellular agriculture might make sense, given that some companies want to co-produce their products rather than contract everything out.

Financing for cellular agriculture startups has primarily come from venture capital and large agribusiness corporations, although some national governments have also invested in these companies (Shieber 2018; Starostinetskaya 2018; Stephens et al. 2019). Publicly and charitably financed research continues at universities, but these efforts have attracted comparatively less funding (Dolgin 2020; Stephens et al. 2019). One engineer with whom we talked said that working with venture capitalists’ tech incubators provided helpful mentoring and a “seal of approval” for a high-risk business. At the same time, significant tradeoffs accompany reliance on such financial entities, such as needing to give up equity share. Several startup representatives explained that the field has already evolved to the point where many new companies would no longer be willing to make that type of tradeoff, and this is a frequent topic of discussion in entrepreneurship circles more broadly. We also heard a major agribusiness representative articulate that cellular agriculture would probably play a complimentary role in the future food system, and that his company had invested in the technology from both a defensive standpoint and a growth standpoint. “[We] will not allow others to disrupt us,” he declared. One startup representative claimed that financial partnerships with major agribusiness companies were mutually beneficial, since the latter could share extensive knowledge on consumer perceptions and purchasing decisions. This could help his startup to make decisions about industry strategy and market engagement, he explained. Despite the current success of the field in raising venture capital, many proponents of cellular agriculture expressed concern that basic research and development would stagnate if initial rounds of funding fail to yield useful patents and/or trade secrets (see also Stephens et al. 2019).

Following political economy of agriculture scholarship (see Brandl and Glenna 2017; Glenna et al. 2015; Kloppenburg 2005, 2010, 2014; Mascarenhas and Busch 2006), we also examined intellectual property and the potential of open innovation. There is nothing inherent to cellular agriculture that requires its myriad processes, components, and intellectual property to be proprietary; the field is a composite of many different disciplines, all of which include open and closed licenses, and numerous organizations are funding and/or developing open source cellular agriculture projects (e.g. Shojinmeat, the Cultivated Meat Modeling Consortium, New Harvest, and the Good Food Institute). Digitalization and synthetic biology are moreover making life sciences research cheaper and more accessible for students, hobbyists, and small companies—groups that frequently lacked the financial and technical resources to do bioengineering in the 1980s and 1990s (Church and Regis 2014; Cumbers and Schmieder 2017; Schwab 2016; Schwab and Davis 2018).

Nonetheless, most cellular agriculture companies remained committed to proprietary licensing strategies, even while conference speakers and participants expressed a spectrum of views on this topic. When answering a question about whether researchers might develop an open source bioreactor, for instance, one startup representative stated, “Well, someone still needs to purchase it, it’s not free.” Another startup representative at the event said that his organization used open source software, because this made the programming process “easier,” while also insisting that they needed to keep their own research proprietary. In contrast, one mechanical engineer said that his startup was very interested in open source collaboration. His company already had a shared “patent pool” with their collaborators, and they used a distributed business model for manufacturing. A company’s approach to open source, he explained, depended on their business model and their unique value proposition. Case in point, a representative at a manufacturing services company said that “in the engineering world” (as opposed to the life sciences), trade secrets and patents are “not as big of a deal.” His company would gladly share their prototypes and templates for cell-based production designs, he said, because people still need to be paid to build it.

We sought to make sense of these companies’ intellectual property strategies by talking with cellular agriculture experts who were not employed by the startups. A biologist confirmed that the startups had been very reluctant to participate in open source research projects, even when they were offered grant money to do so. One investor commented that open source was unlikely to work in cellular agriculture, at least for the foreseeable future, because investors were looking for returns on their contributions, generally through patents and/or competitive advantage. Moreover, he added, in the software world, companies only collaborated through open source on non-essential aspects of their business. To this point, a pharmaceutical representative argued that while cellular agriculture researchers needed to collaborate on best practices, intellectual property and funding silos were still necessary, even if they erected barriers to innovation. We also spoke with an attorney who noted that cellular agriculture investors mostly worked on developing trade secrets as opposed to patents, as the latter required more public disclosure. He further noted that the startups were primarily focused on creating unique production processes as opposed to going out and claiming novel physical objects as their own (as with Monsanto’s rush to patent seeds). Lastly, he argued, if all the companies continued to work independently, without sharing, it could lead to more diverse solutions and approaches, thus preventing a “herd mentality.”

On that note, according to one non-profit representative, her organization’s role was symbiotic with, but distinct from, the startups in the field. While companies focused on “1.0”—with an emphasis on products and investments—her organization focused on “3.0”—with an emphasis on developing future PhDs and chief technology officers, pursuing moonshots, and making incremental advancements. To this end, the non-profit groups have organized conferences, published and funded open research, and called for collaborative research hubs. In sum, however, many attendees at these events remain skeptical that the field will move toward open innovation, because nearly all of the commercial research in the industry is proprietary and beholden to the rapid timeline and outcome demands of venture capital.

### Participation

Although key actors in cellular agriculture articulated skepticism about open approaches to intellectual property and ownership, many advocated for an industry approach that more openly engaged with the general public and potential consumers through participatory communication processes. At New Harvest 2019 and the Good Food Conference 2019 (the latter of which is organized by the Good Food Institute, a non-profit advocacy/techcelerator organization), representatives from Memphis Meats and JUST explicitly called for more broad-based participation and collaboration, both within the industry itself and vis a vis outside communities. As one cellular agriculture executive put it, the “move fast and break things” ethos of Silicon Valley would represent the worst possible way to introduce cell-based foods to the public. Thus far, however, much of this public-facing work (with the exception of engaging the mass media and working with regulators) has been done by non-profit organizations focused on building consumer support. At New Harvest 2019, several startup representatives highlighted the need to bring a wide variety of professions into the emerging industry and reach out to food culture organizations. One startup representative at the event called for “people other than biologists and biomedical engineers” to get involved, although his vision for this involvement was mainly limited to public relations and marketing.

The key question, again, is whether the social organization of cellular agriculture will emulate the existing agribusiness system or whether some of the initial moves towards alternative economic and sociotechnical pathways might become more established. While pursuing their open innovation projects, the non-profits have also celebrated the infusion of venture capital into traditionally structured startup companies (see Specht et al. 2018). For many, the overarching ethical and environmental upside of the technology—particularly with respect to animal protection and climate mitigation—makes this type of traditional business strategy a necessary trade-off (Broad 2019). This is an understandable position, given the urgency and sheer magnitude of these global challenges. Proponents of highly sophisticated meat, dairy, and egg substitutes would argue that these products have moved consumer behavior at least as much as moral appeals to animal rights, although further empirical research is needed to corroborate this (see Broad 2020a, b). The infusion of private capital has moreover grown the industry, given it a sense of momentum, and helped to transition cellular agriculture from a 2000s-era internet joke (Chiles 2013) into a serious technological endeavor (Stephens et al. 2019).

In sum, despite the significant efforts of many motivated organizations and individuals to broaden and deepen participation in cellular agriculture—both within the field and beyond—the continued dominance of traditional ownership structures and technology industry communication strategies has in many ways stymied this work (see also Guthman and Biltekoff 2020). This challenge is compounded by the fact that the software, hardware, and biological specifications used to advance cellular agriculture have effectively been siloed by researchers—they do not share a common set of labels, standards, or datasets that would enable interoperability (Kahan et al. 2020). Moreover, participation has been primarily limited to people with advanced science and technology skills. All of these factors limit the ability of the broader cellular agriculture community to tackle its most significant technical challenges, e.g. developing an affordable growth medium that can be produced at scale (see Specht et al. 2018). To further investigate the potential pathways for an alternative sociotechnical arrangement in cellular agriculture, we explored a second site of study, this time of alternative economic organizations.

## Findings on ownership and participation in alternative economic organizations

Our second site of ethnographic research focuses on four types of alternative economic organization that could be directly used in cellular agriculture: member-owned cooperatives, open-source licensing, social financing, and platform business models. In short, we found that alternative economic organizations seeking to democratize the 4IR are continually navigating between open and closed approaches to ownership and participation. Open ownership and participation facilitate stakeholder inclusivity and diversity, lower barriers to membership, and encourage transparency, profit sharing, democratic governance, crowdsourcing, and open innovation practices. In contrast, closed approaches to ownership and participation involve hierarchical leadership and decision-making, higher barriers to membership for stakeholders who lack the requisite skills or financial resources, lower levels of transparency, a concentration of profit in senior leadership and investors, business models that maintain rigid control over production, and proprietary intellectual property licenses. Organizations have long had to make choices about how they can best balance themselves between these two paradigms (Heimans and Timms 2018), but the increasing risk of a “winner takes all” economy in the 4IR (Galloway 2017) makes this core decision even more critical.

### Ownership

We began our fieldwork for this stage of the project by attending the Linux Foundation’s Open Source Summit 2019. While the Linux Foundation’s original goal was to provide a “neutral home” for open source development of the Linux kernel (the core component of the Linux operating system), it has since expanded to host dozens of open-source community projects by providing “financial and intellectual resources, infrastructure, services, events, and training (Linux Foundation 2020).” As one technology company representative explained to us, this type of legal entity could put cellular agriculture technologies, standards, and licenses into the hands of a “benevolent entity” rather than a single all-powerful company. In doing so, competitors could be brought together to work on projects of shared interest, and the foundation could consolidate and magnify those efforts. The flip side of the coin is that the Linux Foundation is almost entirely sponsored by the world’s biggest tech companies—many of whom are leveraging the power of open software to sell hardware or subscriptions to cloud services, effectively further consolidating their overall market positioning.

In order to explore legal entities that are less dependent on corporate sponsorship, we conducted fieldwork at the National Cooperative Business Association’s Co-Op IMPACT 2019 Conference. Although a cooperative is also a corporation, it is owned by its members or the people who use the services of the cooperative. Members contribute capital and determine the policies of the organization, and each member vote is treated equally. Speakers and participants at Co-Op IMPACT further argued that cooperatives have an added “advantage” with respect to recruitment, retention, and access to a supportive network of cooperative organizations. For one speaker at Co-Op IMPACT, “cooperatives are natural innovators, because innovation occurs in a collaborative environment.” The main challenge, as noted by one speaker at Co-Op IMPACT, is that all successful cooperatives are eventually pressured to conform to traditional business practices (DiMaggio and Powell 1983, p. 147). In agriculture, for example, several large purchasing cooperatives—originally intended to help farmers bargain with processors—have faced member lawsuits for conspiring to fix prices (Douglas 2018).

Regardless of which type of cooperative is being established, according to numerous speakers and participants at Co-Op IMPACT, obtaining financing often proves to be an existential challenge. At the conference, some of the different suggestions for cooperative financing included credit unions, crowdfunding platforms, loan guarantees, cooperative development centers, pooling resources across organizations, federal and philanthropic grants, and seed funding. We explored other potential financing options by conducting fieldwork at SOCAP19, an annual conference dedicated to “social capital markets” and “impact community investing.” Here, speakers and study participants argued that while ownership could be democratized through alternative investment practices, the means by which to do so varies from industry to industry, and investor to investor. Accordingly, they emphasized that it was important for social organizations to understand *who* was investing and *how* they were investing. It was also emphasized that larger scale investment firms could have a place in democratized investment practices, but that it was ultimately up to the individuals within the company to decide whether to invest, and that it often remains a small portion of the company’s portfolios.

When we discussed the prospect of cellular agriculture in this context, a variety of potentially relevant financing options were raised. This included small lenders and community funds (for small business loans); “participatory impact investing,” which facilitates open communication between investors, advisors, and businesses; “blended financing,” which combines multiple strands of funding (e.g. buy-in, in-kind or in-faith investments, or a loan investment with deferred repayment); “direct public offerings” that raise capital by selling debt or equity securities directly to the public, “catalytic capital,” i.e. special funding opportunities for high-risk social investments; employee stock option programs, which allow owners to sell shares and voting power to their workers; and building relationships with funders that also provide technical assistance. Another form of investment highlighted at the event was that of gift and award funding, a competitive process in which private foundations “judge” the intellectual property and business models of applicant organizations.

### Participation

While the legal entity and access to financing provides a base foundation for inclusive innovation, the entity itself is an empty shell without effective participation structures that include and engage people. The most powerful tool in the modern economy for facilitating broad-based and structured participation is arguably the digital platform, since it serves as the business model for the world’s most highly valued companies (Cusumano et al. 2019; Galloway 2017; Lee 2018; Webb 2019), and many of the world’s most successful social movements (Heimans and Timms 2018; Jackson et al. 2020). For the key speakers at Platform Economy Summit Europe 2019 and MIT’s Platform Strategy Summit 2019 (see McAfee and Brynjolfsson 2017; Van Alstyne 2019; Jacobides 2019; Cusumano et al. 2019), digital platforms are at their most effective when they are used to scale broad ecosystems of users, optimize network effects between these users through machine learning, leverage the collective resources of outsiders, and provide unique offerings that could never be developed internally. Transaction platforms (Cusumano et al. 2019), for example, leverage user data to match buyers and sellers with goods and services (including labor and licenses). As outlined by the community leadership workshops at Co-Op IMPACT 2019, Open Source Summit 2019, and Platform Economy Summit Europe 2019, user experience can be further enhanced by contributor ratings, recommendations, news feeds, developer forums, mentor matching, user-friendly interfaces, and social media. Provided that these features are adequately curated, they can help to cultivate shared purpose, network effects, and sustained engagement (see Novkovic 2018; Walter and Lohse 2017). As further noted by speakers at IoT Tech Expo North America 2019, Open Source Summit 2019, and Platform Economy Summit Europe 2019, distributed ledger technologies (i.e. blockchain) can further empower the users of a transaction platform. By providing all platform users with an encrypted digital identity, each user could retain ownership and control over their data, decide when, how, and under what conditions they want that data to be shared, and then use “smart contracts” to automate transactions with other users without having to pay an intermediary (see also Tapscott and Tapscott 2016; Webb 2019).

We investigated another relevant platform strategy at the 2019 Platform Cooperative Consortium in New York City. Here, speakers involved in the creation of the online “Platform Cooperative Development Kit” described their participatory co-design work as grounded in the “anti-Pareto principle.” For these innovators, capitalist technology design was generally focused on the 80% of potential users at the center, whereas their approach focused on the 20% of potential users at the edges. Although this approach is unlikely to supplant the dominant platforms, it might be more inclusive for marginalized communities whose interests and needs are often overlooked. Cellular agriculture might take a similar approach in its development, for example, by outsourcing the central design process and cloud computing to large companies while developing alternative applications for the producers and consumers seeking other ways of operating.

## Synthesis of findings and reflections

Without co-ownership opportunities, shared infrastructures, common standards, and broadly accessible interfaces, it is difficult to facilitate a culture of open innovation. Building such a culture requires strategic organization, namely, a stewarding entity that sees the broader vision, organizes diverse people and processes, and match resources with need. In the field of cellular agriculture, we find that this role could potentially be fulfilled by a non-profit organization, an academic consortium, a member-owned cooperative, or another entity that is not pre-committed to the mandate of its incumbent private funders. For-profit companies such as Integriculture, Memphis Meats, JUST, New Age Meats, and OSPIN are all conducting groundbreaking research to digitize and automate cellular agriculture, but the ability of this work to stimulate external innovation and facilitate co-ownership are likely to be limited by its proprietary context. As a means of synthesizing the insights from our ethnographic findings, we conclude with reflections on potential socio-technical alternatives from the co-production elements of our fieldwork.

### Co-constructing alternative sociotechnical pathways for cellular agriculture

When discussing alternatives to the traditional corporate cellular agriculture industry model, an economist at Co-Op IMPACT 2019 suggested to us the possibility of incorporating a multi-stakeholder cooperative. This type of legal entity could be established as either a for-profit entity or a non-profit foundation, depending on members’ interests. Many of the proponents for cellular agriculture are concerned that deviating from the interests of the startup companies (and their financers) could cause intra-community strife, stymie innovation, and thus impede the field’s ability to address animal and climate-related concerns. A multi-stakeholder cooperative could offer a hybrid solution to this dilemma by assigning the bulk of voting interests to farmers and tech workers, allocating a minority share of voting interests to non-profit organizations and startup companies, and then allowing venture capitalists to participate as non-voting members through a limited cooperative association (LCA) statute. Although the broad spectrum of interests in multi-stakeholder cooperatives are difficult to manage, this strategy would leverage an established legal entity to facilitate co-ownership and participation among many different constituencies. This type of an entity would moreover welcome all stakeholders under a single tent while allocating the majority of the surplus and governing power to farmers and tech workers. Another approach to this dilemma would be to simply incorporate a consumers’ cooperative. This entity would put more ownership value and control over cellular agriculture directly into the hands of the consumers themselves, namely, by lowering prices for retail goods and facilitating petty commodity production at the household level, for instance through 3D printing and/or tabletop bioreactors.

Using the work of one of the Platform Economy Summit Europe speakers as a guide (see Cusumano et al. 2019), we also brainstormed and co-constructed a concept for democratizing participation in cellular agriculture. Here, we envisioned an innovation platform that provided a core infrastructure of digital participation tools for researchers, tech workers, companies, non-profits, farmers, students, gamers, hackers, and ordinary citizens. The unique value proposition of this innovation platform would be a repository of data, software, and hardware specifications—organized and optimized by machine learning—for cellular agriculture development, production, distribution, and consumption. Just as Google’s Open Handset Alliance orchestrates an ecosystem of independent app developers for Android, an innovation platform for cellular agriculture would enable and empower contributors to add their own unique cell lines, datasets, software code, documentation, bioreactor specifications, 3D-printing recipes, ideas for student projects, and other content to the user community. While all of the code for the core innovation platform would be open source, as it is with Android, if contributors wanted to monetize their own spin-offs, they could protect the rights to this work by paying a subscription or royalty fee. As noted by several speakers at Open Source Summit 2019, numerous software companies have found success with this type of dual licensing approach (see also Comino and Manenti 2011; Okoli and Nguyen 2015). Many innovation platforms further empower their ecosystem producers by providing software development kits (SDKs), application programming interfaces (APIs), training, incubators, “virtual machine[s] for development,” “digital foundries” (Cusumano et al. 2019), subsidies for producers, and logistical support (Lee 2018).

Our fieldwork at IoT Tech Expo North America 2019 and Open Source Summit 2019 provided us with additional ideas as to how an innovation platform might distribute power more equitably in the cellular agriculture industry. For example, linking this platform with embedded sensors from participating laboratories, companies, and farms would provide the system with data flow rather than data snapshots. An engineer with whom we spoke confirmed with us that this would be a helpful tool, as it could automate decision-making, logistics, and quality control for smart factories and decentralized supply chains. Farmers, ranchers, and growers could further use these tools to upload and license their genetic materials and other relevant information to the platform, and in exchange they could receive an ownership stake in the organization. Cellular agriculture depends on cell lines from healthy animals, thus ensuring a continued role for livestock farmers, but with an emphasis on significantly fewer animals—to be raised for biodiversity, customer preferences, lifelong husbandry, habitat management, and agritourism as opposed to slaughter volume. Traditional livestock producers and feed growers might also find economic opportunities in this platform ecosystem by leveraging their unique knowledge and expertise in product development, consultation, biosecurity, logistics, supply chains, agribusiness management, and sales (see Hines et al. 2018).

By the same token, the innovation platform’s machine learning tools could provide these farmers with on-demand data for the best times and places that certain types of animals and crops could be raised (Webb 2019). Animal feed growers could also provide a valuable contribution to the cellular agriculture supply chain via feedstock for growth medium (Cosgrove 2017) and scaffolding (Gershlak et al. 2017). Further research, outreach, funding, and infrastructure would be needed to include smaller producers, as they might otherwise lack the capital and training needed to implement the appropriate protocols. In order to be taken seriously, these efforts would need to respect and empower producers’ experiential knowledge, local communities, diverse cultural identities, and economic agency (Klerkx et al. 2019).

Ultimately, the diverse data flows from the innovation and transaction platforms could be standardized and integrated into a single hybrid algorithm, boosted by machine learning, for cellular agriculture research, development, production, processing, distribution, and consumption. When sharing different versions of this organizational vision with study participants, reactions ranged from favorable to mixed. A primary concern was that cellular agriculture companies might still be dissuaded from sharing their intellectual property or even agree on a shared data format, thus making it difficult to design a production system at scale. Others suggested that companies would only be willing to cooperate in the peripheral aspects of their business, that computational biology involves a lot basic research that does not always translate into a financial return, that the methodological diversity in cellular agriculture makes it difficult to aggregate findings across the entire field, and that the life sciences are just too different from software development due to higher levels of data complexity and more barriers to entry. Additional critiques were raised on the basis that companies would need a freemium with a turn-key business model before they would be willing to get involved, and that it would be difficult to get the type of funding that would be necessary to compensate software personnel, pay for computational time, and build for scale. Finally, the high level of regulatory uncertainty looming above the entire sector was also seen as a major barrier to the development of alternative pathways.

At the same time, some of the more positive comments suggested that companies may be willing to work together in the areas of regulatory compliance and basic cell metabolism, that spin-offs from unaffiliated research grants could provide valuable contributions, and that exceptional leadership could drive inclusivity. One engineer with whom we spoke was confident that data sharing agreements could be made with companies, provided that they offered certain restrictions and protections. This has been corroborated by survey and in-depth interview research, which shows that, “firms active in open innovation have a very strong preference for the governance of their open innovation relationships with other firms through formal contracts” (Hagedoorn and Zobel 2015, p. 1). From a technical standpoint, several researchers noted that this type of platform could help to facilitate cell line engineering in silico, develop metabolic maps, and coordinate the activities of biologists, computational biologists, and computer scientists. Two cooperative development specialists at Co-Op IMPACT 2019 described our proposed concept as a “shared services cooperative”—a well-established and successful organizational model that enabled businesses to pool costs for research and development, administration, digital technology, and other “back office services.” Going forward, the business specialists with whom we spoke argued that the core value proposition would need to be quantified (particularly with respect to cost savings for participants and return on investment for funders), carefully licensed, offered up front for free, tested, and iterated. While these comments collectively illustrate the boundaries and limitations of the current study, they also affirm its usefulness by indicating key points of departure for future research and collaboration.

### The Cultured Meat Modeling Consortium: A bridge to the future?

At the time of our fieldwork, the organization that engaged most significantly with the concept of an open innovation platform for cellular agriculture was the Cultured Meat Modeling Consortium (CMMC). At its founding, the CMMC was described as an unincorporated, ad-hoc working group that uses computer aided design to run virtual laboratory experiments on cell fusion, nutrient and oxygen flows to cell tissue, cell proliferation and differentiation, scaffolding, manufacturing costs, and product delivery [CMMC (2019)]. While “the CMMC overall follows an open innovation model, individual projects within the CMMC may take distinct approaches to protect the intellectual property as required by project participants” [CMMC (2019)].

The CMMC’s platform vision is to streamline data collection, as well as standardize and document relevant data on a field-dedicated platform. CMMC’s platform would moreover serve to develop and share open source software tools for collaboration, host open data repositories, and “work with stakeholders to create plans, protocols and workflows to transition data into Systems Biology Markup Languages” (Kahan et al. 2020). This would be a critically important first step towards the more ambitious platform/ecosystem concept that was outlined in the previous section. In order to get an innovation platform up and running, its machine learning program must first be encoded with a basic set of rules to follow. In this case, these rules would be based upon relevant literature and available data in cellular agriculture, engineering and the life sciences (Kahan et al. 2020). With the guidance of these initial rules and datasets, the core digital infrastructure could be further enhanced by a web crawler, i.e., a program that scours the internet for additional knowledge from scientific literature and data according to certain search parameters (Domingos 2015). If this program could moreover be taught to “transfer skills from one domain to the other”—as is done by Baidu’s deep neural net (Webb 2019)—it would enable additional learning and convergence across disciplinary knowledges. Above all, the platform would benefit from developing and facilitating open standards across cellular agriculture scientists and companies, which would help to expedite sharing, research, and implementation (Kahan et al. 2020). Again, machine learning could be used to improve these processes, incorporate additional findings from scientific literatures, recognize patterns, make decisions, and optimize efficiencies beyond human capabilities (Lee 2018). Ultimately, as evidenced by deep learning programs like Alpha Go Zero (Webb 2019), this program could surpass human cognitive abilities to develop cellular agriculture technologies.

Conjoining an innovation platform with a transaction platform could also catalyze the CMMC’s goal of soliciting participation within and beyond the cellular agriculture community [CMMC (2019)]. During our fieldwork, we monitored what the CMMC would choose in terms of its legal entity, which would be critical to its ultimate legacy, as well as its governance, funding, licensing, and civic engagement strategies. Ultimately, the CMMC opted to incorporate as “wholly owned subsidiary” limited liability company that was owned by the founder’s existing social-benefit corporation, Biocellion. Under these terms, commercial subscribers to the CMMC pay a licensing fee for membership, while non-commercial subscribers can join at no cost. Intellectual property is free for non-commercial use, while commercial subscribers can license intellectual property at a cost for any spin-off projects. All profits and fees generated must be used to continue to advance the mission of the CMMC [CMMC (2021)]. While other potential legal entities, including a member-owned cooperative, were considered, the CMMC decided this structure offered the optimal level of flexibility while still aligning with the overall organizational purpose.

## Conclusion

In this paper, we have sought to engage the nascent field of cellular agriculture in conversation with the political economy of agriculture scholarship, namely, on the inescapable question of whether or not this emerging technology will further concentrate wealth and power in the global food system. Innovation without meaningful inclusion has led to inequality, distrust, environmental crises, and social disintegration, and the world’s biggest tech companies are well positioned to continue disrupting and absorbing traditional industries in the coming decades (Galloway 2017). Critically important and valuable innovation, including agroecological approaches to food production, also continues to come from non-industrial contexts (Jasanoff 2016).

To be sure, industrial and non-industrial technologies are not mutually exclusive, and neither is intrinsically better or worse than the other. Both can play a useful and complementary role, depending on the goals and context (Fraser 2020). We would nonetheless conclude that there is a strong case to be made for continued innovation in cellular agriculture, above and beyond the boilerplate argument for protecting animals, reducing antibiotic resistance, and mitigating climate change. The race to develop cellular agriculture is but a microcosm of the broader socioeconomic and geopolitical competition to dominate the 4IR, and the impacts of the Covid-19 pandemic has only further raised the stakes (Chiles 2020). American and Chinese megacorporations are furiously vying with one another to develop dominant platform ecosystems, advanced bioeconomies, and superior artificial intelligence capabilities, while other regions are being left behind (Cumbers and Costa 2020; Lee 2018; McMichael 2020; Staeritz et al. 2020). 4IR technologies are also becoming increasingly affordable and accessible, and strict prohibitions may prove to be practically unfeasible and logistically unenforceable (Cumbers and Schmieder 2017; Church and Regis 2014; Schwab 2016; Schwab and Davis 2018; Webb 2019).

Our findings thus reaffirm the argument for increased investments in 4IR research and education in the public interest (Schwab 2016; Schwab and Davis 2018)—along with a more fundamental reworking of the basic social contract between governments and their citizens in the aftermath of globalization and deindustrialization (King and Le Galès 2017). Government investments in publicly accessible digital infrastructures could help to facilitate a more just transition (Ajena 2019; Mehrabi et al. 2018; Webb 2019), as could public policies that protect platform worker’s rights and consumer privacy (Choudary 2018; Rotz et al. 2019a). Without this type of broad-based, inclusive, and multitiered commitment, democratic societies are likely to lose the 4IR competition to the competing framework of authoritarian capitalism, as China is actively investing billions of dollars into global artificial intelligence research, data collection, business development, infrastructure, and cyberstrategy (Lee 2018; Webb 2019). Stakeholders who are concerned about the “food tech justice” implications of cellular agriculture (Broad 2019) and the 4IR may ultimately find more success by engaging with how these technologies are being developed rather than avoiding them or trying to eradicate them outright.

With this paper, we sought to identify key conditions, indicators, and opportunities that might facilitate more inclusive structures and practices. Our analysis of alternative economic organizations shows that under a certain set of circumstances, *innovation can be inclusive*—through the strategic use of nontraditional legal entities, social financing, open licensing, and structured participation frameworks. *Inclusivity can also enhance innovation*, as a vision and a model for co-ownership and open participation can help to recruit/retain talent and crowdsource problem solving. The continuing work of the CMMC and several different non-profit organizations demonstrates that alternative sociotechnical pathways for cellular agriculture can, and do, exist. Alternative innovation/transaction platforms—up to the point of being owned and governed by a multi-stakeholder cooperative—could allocate significant resources to cellular agriculture startups and their investors while keeping a majority share of the organization’s revenue and voting power in the hands of tech workers and farmers. The financial viability of this concept remains an open question, but given the enormity of the stakes in the 4IR, failing to explore it further would be a tremendous missed opportunity. We would thus encourage political economy of agriculture scholars, agroecologists, and other researchers to similarly consider the potential ways in which digital and 4IR technologies might help to preserve, protect, and expand the global imperative of a just and sustainable food system.

## Abbreviations

4IR: Fourth Industrial Revolution
CMMC: Cultivated Meat Modeling Consortium
